# Complexation and disproportionation of group 4 metal (alkoxy) halides with phosphine oxides†

**DOI:** 10.1039/d4dt01299b

**Published:** 2024-05-28

**Authors:** Carlotta Seno, Rohan Pokratath, Ajmal Roshan Unniram Parambil, Dietger Van den Eynden, Evert Dhaene, Alessandro Prescimone, Jonathan De Roo

**Affiliations:** a Department of Chemistry, University of Basel Mattenstrasse 22 4058 Basel Switzerland Jonathan.DeRoo@unibas.ch

## Abstract

Group 4 Lewis acids are well-known catalysts and precursors for (non-aqueous) sol–gel chemistry. Titanium, zirconium and hafnium halides, and alkoxy halides are precursors for the controlled synthesis of nanocrystals, often in the presence of Lewis base. Here, we investigate the interaction of Lewis bases with the tetrahalides (MX_4_, X = Cl, Br) and metal alkoxy halides (MX_*x*_(OR)_4−*x*_, *x* = 1–3, R = O^i^Pr, O^*t*^Bu). The tetrahalides yield the expected Lewis acid–base adducts MX_4_L_2_ (L = tetrahydrofuran or phosphine oxide). The mixed alkoxy halides react with Lewis bases in a more complex way. ^31^P NMR spectroscopy reveals that excess of phosphine oxide yields predominantly the complexation product, while a (sub)stoichiometric amount of phosphine oxide causes disproportionation of the MX_*x*_(OR)_4−*x*_ species into MX_*x*+1_(OR)_3−*x*_ and MX_*x*−1_(OR)_5−*x*_. The combination of complexation and disproportionation yields an atypical Job plot. In the case of zirconium isopropoxy chlorides, we fitted the concentration of all observed species and extracted thermodynamic descriptors from the Job plot. The complexation equilibrium constant decreases in the series: ZrCl_3_(O^i^Pr) > ZrCl_2_(O^i^Pr)_2_ ≫ ZrCl(O^i^Pr)_3_, while the disproportionation equilibrium constant follows the opposite trend. Using calculations at the DFT level of theory, we show that disproportionation is driven by the more energetically favorable Lewis acid–base complex formed with the more acidic species. We also gain more insight into the isomerism of the complexes. The disproportionation reaction turns out to be a general phenomenon, for titanium, zirconium and hafnium, for chlorides and bromides, and for isopropoxides and *tert*-butoxides.

## Abstract 2

Metal alkoxy halides of group 4 interact with Lewis bases, causing disproportionation and complexation.

## Graphical abstract

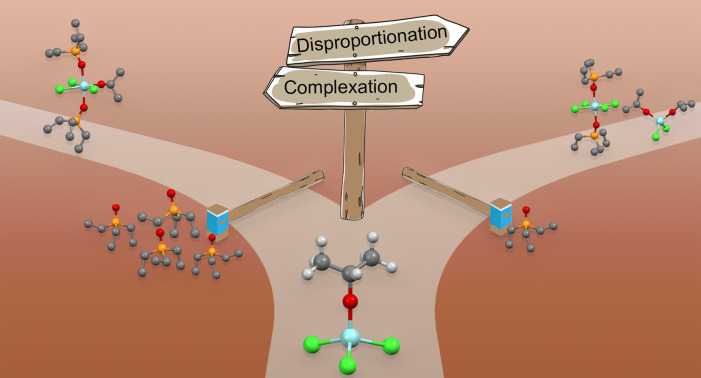

## 1. Introduction

Lewis acids play an essential role in many catalytic and biological processes and understanding their interaction with Lewis bases has driven chemistry forward. Group 4 metals (Ti, Zr, Hf) produce an interesting class of Lewis acids, almost exclusively featuring the +IV oxidation state.^1^ The metal halides are economical precursors for the synthesis of group 4 materials, ranging from amorphous gels,^2,3^ to crystalline nanoparticles.^4–8^ Given that the halides are often dissolved in alcohol or combined with metal alkoxides, the actual reactive species in solution are often mixed alkoxy halides.^9–11^ In the synthesis of zirconia nanocrystals from zirconium chloride and zirconium isopropoxide, zirconium di-isopropoxy di-chloride is a confirmed intermediate, which interacts with the Lewis base present: tri-*n*-octylphosphine oxide (TOPO). The formed complex decomposes at 340 °C to zirconia nanocrystals.^11–13^1
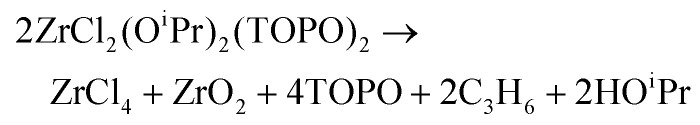


The same chemistry is relevant for titania and hafnia nanocrystals, where the choice of metal halide, metal alkoxide, and of the Lewis base plays a crucial role in the determination of the final properties of the product.^4,14–18^ Although the nature and stability of the formed metal complexes are of direct consequence to the outcome of nanocrystal syntheses, detailed insights into the coordination of alkoxy halides are lacking.

The metal halides were more extensively studied. All twelve possible MX_4_ compounds are known, with M = Ti, Zr, Hf and X = F, Cl, Br, I.^1^ All of them are strong Lewis acids. Some are monomeric molecules (*e.g.*, TiCl_4_, TiBr_4_ and TiI_4_), while others form polymeric solids (TiF_4_, ZrCl_4_ and HfCl_4_).^19–21^ The polymeric structure consists of bridging halides, which increases the coordination number. It is preferred for the larger metals (Zr/Hf) and/or smaller halides (F).^7^ The metal halides react with Lewis bases (L), forming the MX_4_L_2_ adducts, with a coordination number of 6.^22^ In case of titanium fluoride, the solution behavior is often complex with the ionic species TiF_3_L_3_^+^ and Ti_4_F_18_^2−^ present, next to TiF_4_L_2_.^22^ Under-supply of Lewis base (*e.g.*, only one equivalent) further promotes ionization:

2 3TiF_4_L → TiF_3_L_3_^+^ + Ti_2_F_9_^−^

The isolated species in the solid state is usually *cis*-TiF_4_L_2_. A mixture of *trans*-TiF_4_L_2_ and *cis*-TiF_4_L_2_ complexes were found in solution in case the Lewis base was sufficiently steric. While triphenylphosphine oxide produces *cis*-TiF_4_L_2_ in the solid state, 2,6-dimethylpyridine-*N*-oxide produces the *trans* configuration. The *cis*/*trans* equilibrium was rationalized as follows.^23^

• Steric repulsion between halides favors the *trans* configuration

• Steric repulsion between the ligands favors the *trans* configuration

• To maximize halide-metal p_π_–d_π_ bonding, the *cis* configuration is favored.

The last argument is justified by the shorter bond length of the halide *trans* to the Lewis base, compared to the two fluorides *trans* with respect to each other. The halide competes more effectively for the d_π_ with a neutral Lewis base than with another halide. While the above arguments were originally made for titanium fluoride, they explain a more general pattern in the literature.

The halide effect is apparent from the THF complexes of TiF_4_ and TiCl_4_. TiF_4_(THF)_2_ has the *cis* configuration,^24^ but TiCl_4_(THF)_2_ is found both as *cis* and *trans*.^25,26^ For a larger metal, the steric repulsion between the chloride ligands is mitigated, evidenced by the sole isolation of *cis*-ZrCl_4_(THF)_2_ and *cis*-HfCl_4_(THF)_2_.^27,28^ Using tetramethylethylenediamine as bidentate Lewis base, *cis*-ZrCl_4_L_2_ was obtained and again, the Zr–Cl bond distances are longer for the two *trans* chloride ligands (2.43 Å) and shorter for the chloride ligands *trans* to the Lewis base (2.41 Å).^29^ Acetonitrile (ACN) is a less steric ligand than THF and only *cis* configurations were determined for TiCl_4_(ACN)_2_,^30,31^ TiBr_4_(ACN)_2_,^32^ ZrCl_4_(ACN)_2_,^33,34^ and ZrBr_4_(ACN)_2_.^35,36^ On the other hand, tri-*n*-octylphosphine oxide is more sterically bulky than THF and favors the *trans* configuration for ZrCl_4_(R_3_PO)_2_.^11^

Ligand exchange of Lewis bases bound to TiF_4_ was shown to occur *via* a dissociative mechanism featuring an intermediate with coordination number of 5.^22,37,38^ The kinetic stability of the complexes follows the order of Lewis base strength^39^3
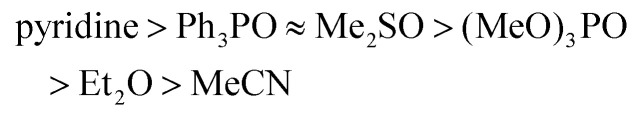


Mixed alkoxy halide species are formed when a metal halide is mixed with a metal alkoxide.^2,40,41^ The stoichiometry of the final product can be varied:

4 3MCl_4_ + M(OR)_4_ → 4MCl_3_(OR)

5 MCl_4_ + M(OR)_4_ → 2MCl_2_(OR)_2_

6 MCl_4_ + 3M(OR)_4_ → 4MCl(OR)_3_

The mixed alkoxy chloride also forms by reacting the metal alkoxide with acetyl chloride.^42^ The ester is formed as a by-product.

7 M(OR)_4_ + *x*RCOCl → MCl_*x*_(OR)_4−*x*_ + *x*RCOOR

The distinction between the different MCl_*x*_(OR)_4−*x*_ complexes *via*^1^H NMR is highly challenging due to the complex structure of the resonances.^2,40^ After complexation with phosphine oxides, the different species can be distinguished *via*^31^P NMR.^11^ However, the Lewis acid behavior of these mixed halide alkoxides is largely unexplored.

Here, we gain insight into the interaction between group 4 MCl_4−*x*_(OR)_*x*_ Lewis acids (M = Ti, Zr or Hf) and phosphine oxide Lewis bases, relevant for nanocrystal syntheses. Focusing first on pure metal halides, we synthesize and analyze the structure of previously unreported ZrBr_4_(THF)_2_ and various MX_4_(TPPO) (TPPO = triphenylphosphine oxide, X = Cl or Br) complexes with single-crystal XRD. All obtained complexes feature the *trans* configuration of the Lewis bases. Second, we study the interaction of mixed isopropoxy chloride with tri-*n*-octylphosphine oxide using ^31^P NMR spectroscopy. We observe both complexation and disproportionation reactions. The latter are especially prevalent at sub-stoichiometric amounts of Lewis base and this behavior highly complicates the Job plot. Focusing on the case of zirconium, we model the Job plot using a set of chemical equations (in COPASI^43^), thus extracting the equilibrium constants for both complexation and disproportionation. With decreasing Lewis acidity (MCl_3_(OR) > MCl_2_(OR)_2_ > MCl(OR)_3_), we find a lower complexation constant but a higher propensity for disproportionation. We showed the generality of the disproportionation behavior for all three metals, for different halides and different alkoxides.

## 2. Results and discussion

### 2.1. Lewis base adducts of MCl_4_

**Fig. 1:**
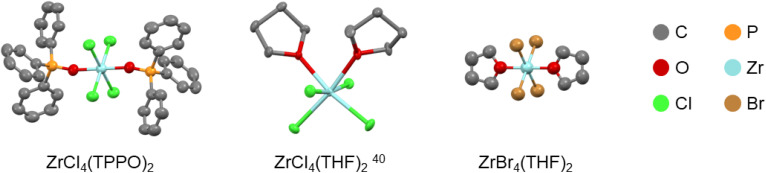
Structures of zirconium halide complexes determined by single-crystal XRD. The hydrogen atoms are omitted for clarity. Table S2† reports the resolved crystallographic parameters.

The Lewis base adducts of titanium tetrahalides are well-reported in the literature but zirconium and hafnium tetrahalides are less well-documented. For completion, we synthesized and crystallized *trans*-ZrBr_4_(THF)_2_, see Fig. 1. The bond distances are reported in Table S1,† together with selected structures from the literature. While ZrCl_4_(THF)_2_ is *cis*, ZrBr_4_(THF)_2_ has the *trans* configuration. The M–O bond distance is slightly shorter in ZrBr_4_(THF)_2_, which is likely due to the absence of steric repulsion between the two THF ligands since the same effect is observed for *cis*-TiCl_4_(THF)_2_ and *trans*-TiCl_4_(THF)_2_, see Table S1.†

We previously showed that phosphine oxides quantitatively displace THF from zirconium chloride.^11^ Therefore, we synthesized the triphenylphosphine oxide (TPPO) complexes of ZrCl_4_, ZrBr_4_ and HfCl_4_ by ligand exchange from the THF complex and single-crystals were grown.

8 MX_4_(THF)_2_ + 2TPPO → MX_4_(TPPO)_2_ + 2THF

Structural analysis *via* single-crystal XRD indicates that the TPPO complexes have the *trans* configuration, see Fig. 1 for the structure of *trans*-ZrCl_4_(TPPO)_2_ (the other structures are shown in Fig. S1†). Including literature data, we conclude that all TPPO adducts of zirconium and hafnium tetrahalides adopt the *trans* configuration (Table 1). Compared to the THF complexes, the TPPO complexes generally feature a longer M–X distance and a shorter M–O distance (Table S1†). This is a reflection of the higher Lewis basicity of phosphine oxides. In general, Table 1 follows the general trend laid out in the introduction. Larger halides and ligands promote the *trans* configuration. In addition, a larger metal size promotes the *cis* configuration as it allows to alleviate steric repulsion between the halides. A notable exception to this trend is the contrast between *cis*-TiF_4_(TPPO)_2_ and *trans*-ZrF_4_(TPPO)_2_. Even though the zirconium atom is larger than titanium, the configuration switches from *cis* to *trans*. We hypothesize that the energy difference between the fluorine p-orbitals and the zirconium d-orbitals is too high to allow for sufficient interaction, thereby removing the driving force for the *cis* configuration. All synthesized complexes were characterized by ^1^H and ^31^P NMR spectroscopy (Fig. S2†), thermogravimetric analysis (Table S3†) and infrared spectroscopy (Fig. S3†). Powder XRD confirmed that the bulk material has the same structure as the single crystals (Fig. S4†). The triphenylphosphine oxide complexes are all poorly soluble in chloroform and effectively insoluble in benzene, but ligand exchange for tri-*n*-octylphosphine oxide (TOPO) generates soluble complexes, with ^31^P NMR shifts consistent with previous assignments (Fig. S5–S7†).^11^ TiCl_4_(TOPO)_2_ appears as a sharp singlet at 77.4 ppm, ZrCl_4_(TOPO)_2_ at 72.9 ppm and HfCl_4_(TOPO)_2_ at 73.0 ppm. The Gutmann-Beckett method ranks the effective Lewis acidity by the ^31^P NMR shift of triethylphosphine oxide (TEPO) bound to a Lewis acid.^44,45^ A high chemical shift is related to a high effective Lewis acidity. Given the similar structure of TEPO and TOPO, we conclude that TiCl_4_ has a higher effective Lewis acidity than ZrCl_4_ and HfCl_4_. The latter two have about the same effective Lewis acidity.

**Table 1:** Overview of *cis*/*trans* configuration in different group 4 metal halide complexes with either tetrahydrofuran (THF) or triphenylphosphine oxide (TPPO) as Lewis base

	THF	TPPO
TiF_4_	*cis* ^24^	*cis* ^24^
TiCl_4_	*cis* ^25^ and *trans*^26^	*trans* a ^46^

ZrF_4_	—	*trans* ^47^
ZrCl_4_	*cis* ^27^	*trans* a
ZrBr_4_	*trans* a	*trans* a

HfCl_4_	*cis* ^28^	*trans* a

a This work.

### 2.2. Complexation and disproportionation of ZrCl_*x*_(O^i^Pr)_4−*x*_

**Fig. 2:**
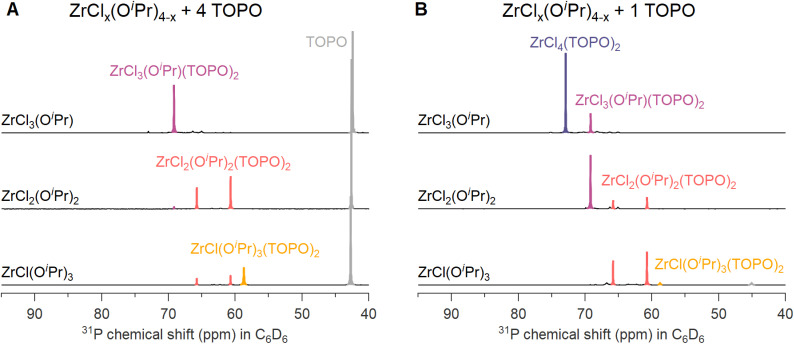
^31^P NMR spectra at room temperature of zirconium alkoxy chloride complexes MCl_*x*_(O^i^Pr)_4−*x*_ in benzene-d_6_ with either (A) 4 equivalents or (B) 1 equivalent of TOPO. The concentration of zirconium is 0.05 M when mixed with 4 equivalents, and 0.125 M with 1 equivalent.

We synthesized three ZrCl_*x*_(O^i^Pr)_4−*x*_ (*x* = 1–3) compounds by reacting Zr(O^i^Pr)_4_·^i^PrOH with acetylchloride in the correct ratios, see eqn (7). We then added an excess (4 equivalents) of TOPO to each compound to assess the complexation behavior. In case of MCl_3_(O^i^Pr), the ^31^P NMR spectrum (Fig. 2A) shows one main signal for MCl_3_(O^i^Pr)(TOPO)_2_. In the case of MCl_2_(O^i^Pr)_2_, we observe two resonances pertaining to 2 isomers, see further details in the computational section. In case of MCl(O^i^Pr)_3_, we observe a less intense signal for the MCl(O^i^Pr)_3_(TOPO)_2_ complex and also the resonances of the two MCl_2_(O^i^Pr)_2_(TOPO)_2_ isomers are present. Based on the assignment and the positions in ^31^P NMR spectrum, the effective Lewis acidity is ranked: ZrCl_4_ > ZrCl_3_(O^i^Pr) > ZrCl_2_(O^i^Pr)_2_ > ZrCl(O^i^Pr)_3_.

When only 1 equivalent of TOPO is added, the speciation changes, see Fig. 2B. Starting from MCl_3_(O^i^Pr), we detect MCl_4_(TOPO)_2_ as the main species in the ^31^P NMR spectrum. For MCl_2_(O^i^Pr)_2_, we retrieve mostly MCl_3_(O^i^Pr)(TOPO)_2_ and for MCl(O^i^Pr)_3_, we find almost exclusively the two isomers of MCl_2_(O^i^Pr)_2_(TOPO)_2_. Note that species, which are not coordinated by TOPO, are not detected by ^31^P NMR. To explain the data, we hypothesize a disproportionation reaction and formation of the stronger Lewis acid–base adduct. Take the example of ZrCl_3_(O^i^Pr). The reaction with 1 equivalent of TOPO can be split in a disproportionation (eqn (9)) and a complexation reaction (eqn (10)):9
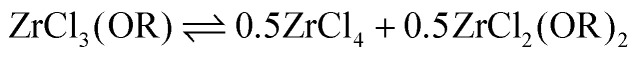
10
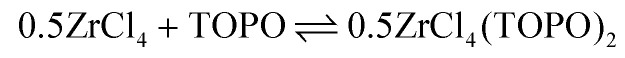


Giving the total:11
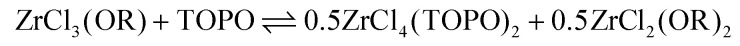


After disproportionation, the Lewis base forms an adduct with the most Lewis acidic species, ZrCl_4_. Hence, ZrCl_4_(TOPO)_2_ is detected in NMR, while ZrCl_2_(OR)_2_ is undetectable by ^31^P NMR. In the absence of a disproportionation, complexation could only result in either 1 equivalent of ZrCl_3_(OR)(TOPO) or in 0.5 equiv. ZrCl_3_(OR)(TOPO)_2_ and 0.5 equiv. ZrCl_3_(OR). We thus assign the driving force of the disproportionation to the formation of stronger Lewis acid–base adduct; ZrCl_4_(TOPO)_2_. When supplying an excess of Lewis base, the disproportionation pathway is suppressed, the extent of which depends on the alkoxy chloride. For the case of MCl(O^i^Pr)_3_, the MCl_2_(O^i^Pr)_2_(TOPO)_2_ isomers (*i.e.*, the disproportionation product) were still significantly present at 4 equivalents of TOPO, see Fig. 2A.

There is no literature precedent for ligand-induced disproportionation in group 4. However, TiCl_3_(OMe) disproportionates upon heating and cannot be purified by distillation:^48^12
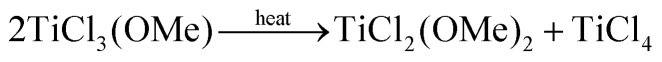


Precedent for ligand-induced disproportionation is found in group 13. Wiberg and coworkers reported in 1935 that BCl_*x*_(OR)_3−*x*_ species disproportionate into BCl_*x*+1_(OR)_3−*x*−1_ and BCl_*x*−1_(OR)_3−*x*+1_ (with *x* = 1, 2) when trimethylamine or an ether is introduced.^49,50^ A quantitative analysis of the phenomenon was not reported.

### 2.3. Quantitative fit of the Job plot

**Fig. 3:**
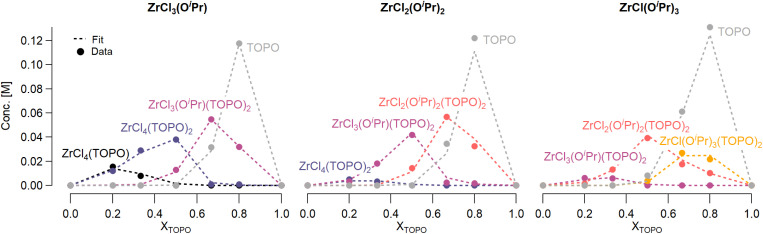
Job plots of ZrCl_3_(O^i^Pr), ZrCl_2_(O^i^Pr)_2_ and ZrCl(O^i^Pr)_3_. The dots represent the data and the dashed lines the quantitative fit. Various species formed during the reaction are indicated and plotted against the mole fraction of TOPO (X_TOPO_). The concentration of each species was calculated by integrating the corresponding peak from the ^31^P NMR, see ESI.†

Job plots are a popular technique to determine the stoichiometry of binding events.^51–54^ In such a plot, the relative mole fraction of reagents is varied while keeping the sum of the concentrations constant. In case of zirconium isopropoxy chlorides interacting with TOPO, the Job plot becomes highly complicated since the reaction is not limited to complexation but also features disproportionation, see Fig. 3.

To quantify the disproportionation observed in the previous section, we modeled the Job plots with a series of equilibrium reactions. For the case of ZrCl_3_(O^i^Pr), we take a set of three equilibria, composed of a complexation (eqn (13)), a disproportionation (eqn (14)) and a decomplexation equilibrium (eqn (15)), where L = TOPO.13
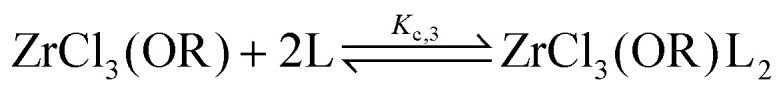
14
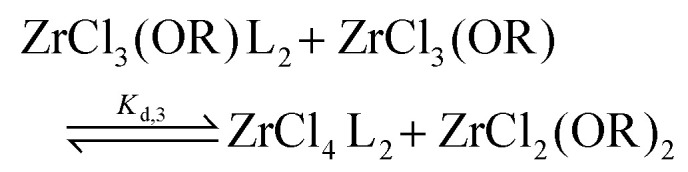
15
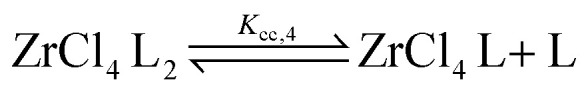


For the Job plots of ZrCl_2_(O^i^Pr)_2_ and of ZrCl(O^i^Pr)_3_ we used a different set of reactions. Eqn (16) and (19) represent the complexation of the respective alkoxy chloride complex with TOPO. This is complemented by two disproportionations eqn (17) and (18) for ZrCl_2_(O^i^Pr)_2_, and eqn (20) and (21) for ZrCl(O^i^Pr)_3_. The second disproportionation equation has been added in each case since the NMR spectrum of ZrCl_2_(O^i^Pr)_2_ features the presence of ZrCl_3_(O^i^Pr)(TOPO)_2_ and ZrCl_4_(TOPO)_2_ when TOPO is added. Similar observations are made for ZrCl(O^i^Pr)_3_.16
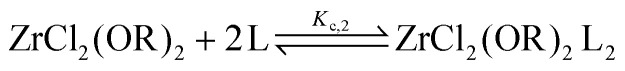
17
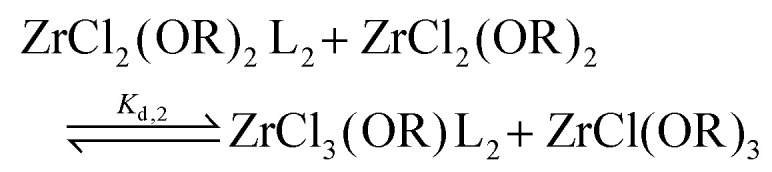
18
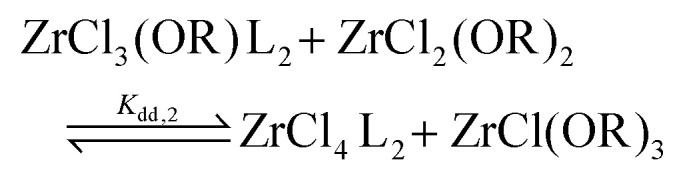
19
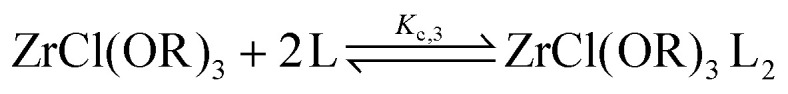
20
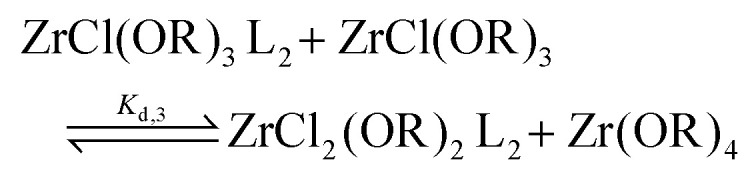
21
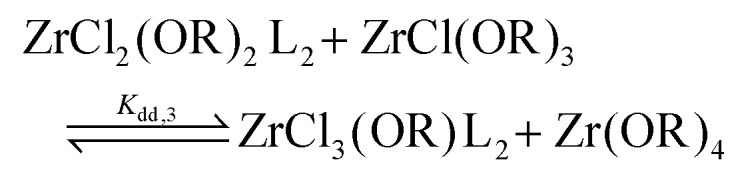


For all three cases, we obtain excellent fits to the data (Fig. 3 and details in the ESI†). The equilibrium constants are reported in Table 2. The complexation constant (*K*_c,*x*_) decreases with decreasing chloride content but the trend is not linear. *K*_c,*x*_ is slightly higher for ZrCl_3_(O^i^Pr) than for ZrCl_2_(O^i^Pr)_2_, but drops two orders of magnitude for ZrCl(O^i^Pr)_3_, which might be due to steric effects of the isopropoxides in addition to a decreasing Lewis acidity. The opposite trend is found for the disproportionation constant (*K*_d,*x*_), which is statistically the same for ZrCl_3_(O^i^Pr) and ZrCl_2_(O^i^Pr)_2_ but increases for ZrCl(O^i^Pr)_3_. This is likely due to the larger difference in complexation strength between ZrCl_2_(O^i^Pr)_2_ and ZrCl(O^i^Pr)_3_ compared to the difference between ZrCl_3_(O^i^Pr) and ZrCl_2_(O^i^Pr)_2_.

**Table 2:** Equilibrium constants for the sets of equilibrium reactions that describe the complexation and disproportionation mechanisms of the three zirconium alkoxy chloride complexes

	ZrCl_3_(O^i^Pr)	ZrCl_2_(O^i^Pr)_2_	ZrCl(O^i^Pr)_3_
*K*_c,*x*_	9.1 × 10^6^ ± 6.9 × 10^6^	1.6 × 10^6^ ± 1.3 × 10^6^	4.6 × 10^4^ ± 2.6 × 10^4^
*K*_cc,4_	1.3 × 10^−5^ ± 0.6 × 10^−5^		
*K*_d,*x*_	13.9 ± 3.2	14.5 ± 5.8	116.9 ± 63.5
*K*_dd,*x*_		0.07 ± 0.01	0.16 ± 0.02

### 2.4. Computational insights

**Fig. 4:**
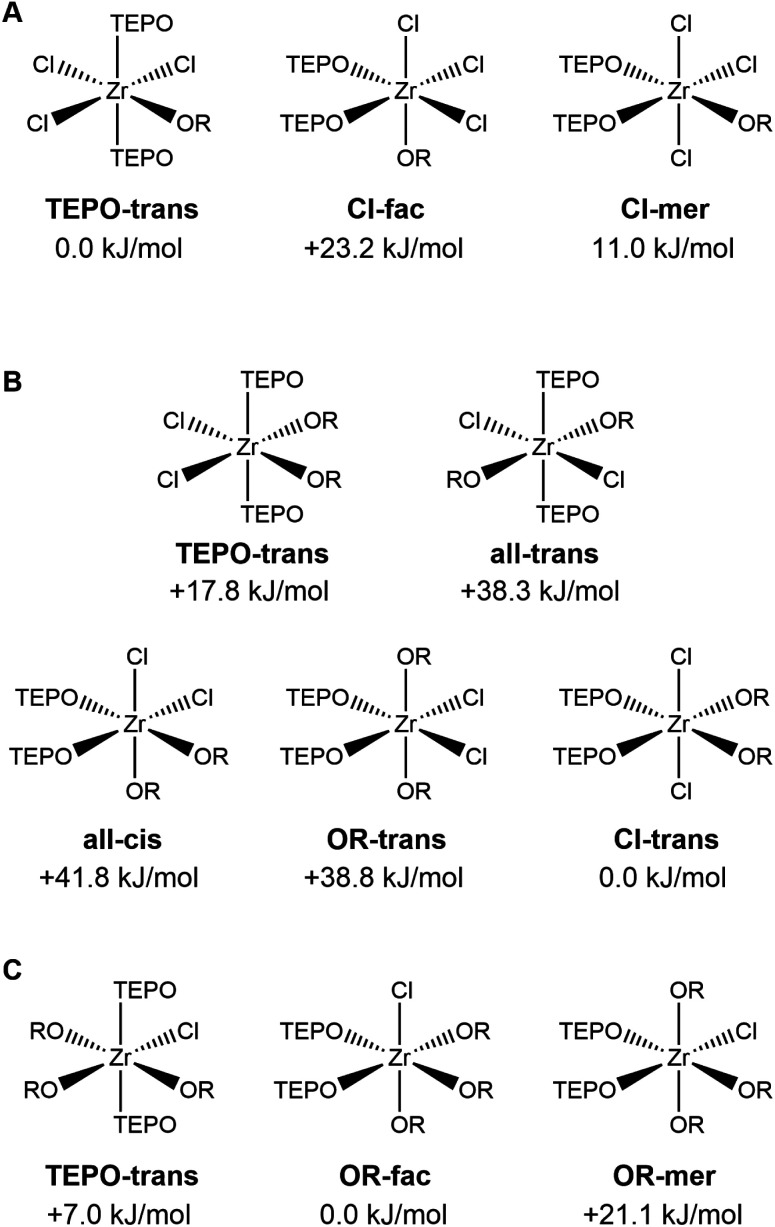
The different possible isomers for the ZrCl_*x*_(O^i^Pr)_4−*x*_(TEPO)_2_ complexes (*x* = 1–3). The relative energy compared to the most stable isomer is indicated.

We sought to compare our experimental data with computations and further strengthen our hypothesis for the disproportionation driving force. Following our earlier established method based on DFT (see Experimental section), we optimized the structures of ZrCl_*x*_(O^i^Pr)_4−*x*_ (*x* = 1–4) and their complexes with THF or with triethylphosphine oxide (TEPO). TEPO is computationally less demanding than TOPO and forms identical complexes, as shown in Fig. S11.† All optimized structures are available as structure files in the ESI.† We considered all isomers for both THF and TEPO complexes, optimized their geometry, and calculated their energy, see Fig. 4 for TEPO (and Fig. S12† for THF). For all THF complexes, we find that the lowest energy isomer has the THF ligands in the *cis* configuration, consistent with the single-crystal data of ZrCl_4_(THF)_2_. The TEPO complexes show surprising trends. The ZrCl_3_(O^i^Pr)(TEPO)_2_ complex is most stable in the TEPO-*trans* configuration (Fig. 4), again consistent with the single-crystal data of ZrCl_4_(TPPO)_2_. However, the ZrCl_2_(O^i^Pr)_2_(TEPO)_2_ complex prefers the Cl-*trans* isomer, with the TEPO ligands *cis* to each other. This configuration is most effective in reducing the steric interactions between isopropoxides and TEPO ligands. It is interesting to examine the structure of the Cl-*trans* isomer. The octahedral coordination is distorted, with the Cl–Zr–Cl bond angle measuring 166 degrees and the chloride atoms pushed towards the TEPO side. The alpha carbon of the isopropoxide ligands (*i.e.*, the branching point or the origin of sterical hindrance) is located at 3.19 and 3.34 Å from the central Zr atom. This makes isopropoxide a more steric ligand than TEPO since the Zr–P distance is longer: 3.50 and 3.68 Å (for TEPO, phosphorus is the branching point). The ZrCl(O^i^Pr)_3_(TEPO)_2_ complex prefers the OR-fac isomer (Fig. 4), again with the TEPO ligands in the *cis* configuration, and the octahedral coordination distorted with chloride being pushing towards TEPO. The TEPO-*trans* isomer is only 7 kJ mol^−1^ less stable though.

Using the calculated energies, we computed the enthalpy changes for the (gas-phase) complexation reactions (Table 3), which represent the global Lewis acidity since the latter is defined as the thermodynamic tendency to form Lewis acid–base pairs.^55^ All reactions are exothermic and the complexation with TEPO is favored over complexation with THF, in agreement with experimental results. The complexation reaction becomes less exothermic in the series: ZrCl_4_ > ZrCl_3_(O^i^Pr) > ZrCl_2_(O^i^Pr)_2_ > ZrCl(O^i^Pr)_3_. This trend in global Lewis acidity agrees with the trend in effective Lewis acidity, given by the ^31^P NMR shifts. The calculated values of Δ*H* cannot be directly compared to the experimental equilibrium constants since the latter depend on Δ*G*. For example, for ZrCl_3_(O^i^Pr), the experimental equilibrium constant translates in Δ*G* = −39.7 kJ mol^−1^. The large theoretical value of Δ*H* is thus likely offset by an unfavorable change in entropy, as one would expect for an association equilibrium. Furthermore, the uncoordinated Lewis acid is a tetrahedral monomer in the calculations, and thus dimerization (or polymerization in case of ZrCl_4_) may account for discrepancies with experimental results.^55^ As a final disclaimer, the calculations were performed in the gas phase and no solvent effects were taken into account.

**Table 3:** Calculated changes in enthalpy for the complexation reaction of the different zirconium isopropoxy chlorides with either tetrahydrofuran (THF) and triethylphosphine oxide (TEPO). The lowest energy isomer was used for the calculation

	Δ*H* (kJ mol^−1^)	
	L = THF	L = TEPO
ZrCl_4_ + 2L ⇌ ZrCl_4_L_2_	−117.1	−235.0
ZrCl_3_(O^i^Pr) + 2L ⇌ ZrCl_3_(O^i^Pr)L_2_	−105.3	−188.0
ZrCl_2_(O^i^Pr)_2_ + 2L ⇌ ZrCl_2_(O^i^Pr)_2_L_2_	−90.3	−177.4
ZrCl(O^i^Pr)_3_ + 2L ⇌ ZrCl(O^i^Pr)_3_L_2_	−65.3	−113.6

When comparing the experimental data of ZrCl_2_(O^i^Pr)_2_(TEPO)_2_ and the theoretical calculations, we are faced with a discrepancy. Two peaks are observed in the ^31^P NMR spectrum and we previously assigned them to the TEPO-*trans* and the all-*trans* isomers, without considering the other isomers.^11^ From our current calculations, Cl-*trans* isomer is clearly preferred. This is not an effect of the chain length since the two peaks appear in the same ratio for both TEPO and TOPO. The two signals always behave as one and we did not observe one without the other. We discarded the hypothesis that the two resonances represent the two phosphine oxide ligands in the same complex since the integrals are not 1 : 1 but 0.35 : 0.65. Our current best hypothesis is that the TEPO-*trans* structure is more favorable than we currently predict by DFT. The existence of multiple isomers in solution could also explain the occurrence of many more resonances in deuteroform compared to benzene (for a given composition and stoichiometry).^11^ It also points to the importance of the solvent in stabilizing certain isomers.

While acknowledging the above limitations, we computed Δ*H* for the spontaneous disproportionation reaction and for the Lewis base-induced disproportionation, see Table 4. The disproportionation in the absence of Lewis bases is quite endothermic (entries 1 and 2). However, when we compute Δ*H* for the TEPO-assisted disproportionation, the reactions become more favorable (entries 3 and 4). These calculations correspond to eqn (17) and (20) and the experimental equilibrium constants were reported in Table 2. When calculating Δ*G* from *K*_d,3_, we obtain −6.5 kJ mol^−1^, significantly smaller than the computed enthalpy change (−29 kJ mol^−1^). We attribute this to the presence of weak Lewis bases in the reaction mixture. Indeed, it is known that esters produced during the synthesis of the mixed alkoxy chlorides can coordinate to the metal center (see eqn (7)). The effect of the presence of a weaker Lewis base (*e.g.*, THF), can be also theoretically computed, see entries 5 and 6 in Table 4. It is clear that the driving force for disproportionation decreases when THF is available to coordinate the Lewis acid sites which are not coordinated by phosphine oxide. This is reproduced experimentally for ZrCl_3_(O^i^Pr), see Fig. S13.†

**Table 4:** Calculated changes in enthalpy for the disproportionation reaction of the different zirconium isopropoxy chlorides with L = TEPO. The lowest energy isomer was used for the calculation

Entry	Equilibrium	Δ*H* (kJ mol^−1^)
1	2ZrCl_3_(O^i^Pr) ⇌ ZrCl_4_ + ZrCl_2_(O^i^Pr)_2_	18.0
2	2ZrCl_2_(O^i^Pr)_2_ ⇌ ZrCl_3_(O^i^Pr) + ZrCl(O^i^Pr)_3_	16.9

3	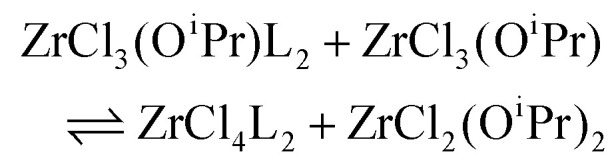	−29.0
4	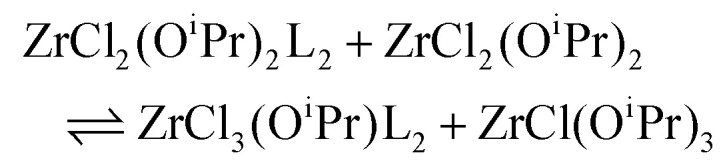	6.3

5	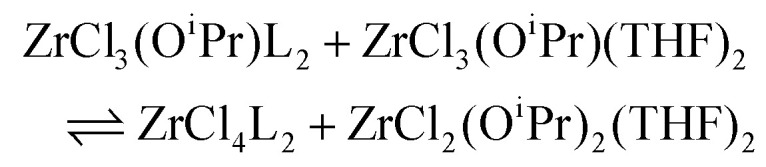	−13.9
6	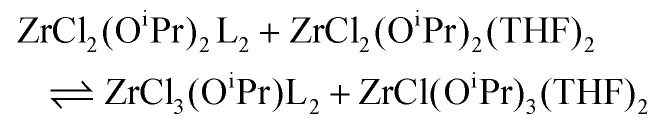	31.2

### 2.5. Generalization

**Fig. 5:**
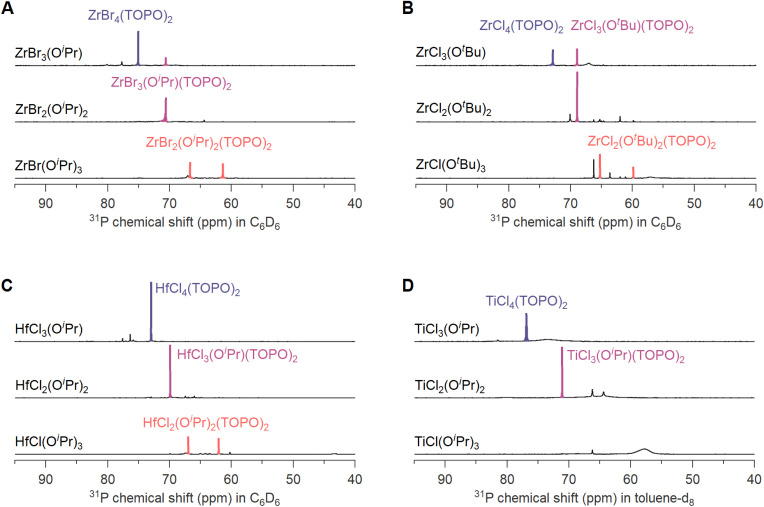
^31^P NMR spectra of (A) zirconium isopropoxy bromide (B) zirconium *tert-*butoxy chloride (C) hafnium isopropoxy chloride (D) titanium isopropoxy chloride complexes mixed with 1 equivalent of TOPO. In case of A and B, the alkoxy halides were synthesized from the respective zirconium halide THF complex.

To test the generality of the disproportionation behavior, we also investigated other alkoxy halides, varying the metal, the alkoxide and the halide. Fig. S14–S17† show the NMR spectra with 2 or 4 equivalents TOPO, to identify the complexation products. Fig. 5 shows the spectra with one equivalent of TOPO and in all four cases, the disproportionation products are detected. We observe some interesting differences. In case of zirconium isopropoxy tribromide, we notice a stronger tendency for disproportionation compared to the above zirconium isopropoxy trichloride in the presence of THF (Fig. S13†). We synthesized the alkoxy bromide by mixing ZrBr_4_(THF)_2_ and Zr(O^i^Pr)_4_(^i^PrOH) due to the low solubility of ZrBr_4_. In case of zirconium *tert-*butoxy trichloride (synthesized from ZrCl_4_(THF)_2_ and Zr(O^*t*^Bu)_4_) we observe a stronger disproportionation compared to zirconium isopropoxy trichloride, but weaker than zirconium isopropoxy tribromide. The hafnium isopropoxy chlorides are synthesized from HfCl_4_ and Hf(O^i^Pr)_4_(^i^PrOH) so there is no THF or ester present, only 0.25 equivalents of isopropanol. A complete disproportionation is the result. In case of titanium, we observe the expected disproportionation products for TiCl_3_(O^i^Pr) and TiCl_2_(O^i^Pr)_2_, but not for TiCl(O^i^Pr)_3_. As demonstrated in Fig. S17,† TiCl_4_(TOPO)_2_ and TiCl_3_(O^i^Pr)(TOPO)_2_ are detectable species with narrow resonances in the ^31^P NMR spectrum, but TiCl_2_(O^i^Pr)_2_(TOPO)_2_ is not detectable, featuring very broad lines, indicative of chemical exchange between bound and free TOPO.

## 3. Conclusions

We studied the interaction of group 4 metal halides and metal alkoxy halides with Lewis bases. We found that phosphine oxides are excellent hard bases for these types of Lewis acids and form Lewis acid–base adducts with a 1 : 2 stoichiometry. Sub-stoichiometric amounts of Lewis bases lead to disproportionation of the metal alkoxy halides. We were able to extract the experimental equilibrium constants for both complexation and disproportionation equilibria from the Job plot. The complexation equilibrium constant decreases with the Lewis acidity of the complex, *i.e.* with the chloride content. Calculations at the DFT level of theory confirmed the trend and allowed us to rationalize the driving force for disproportionation: the formation of more stable Lewis acid–base adducts. These results thus provide fundamental insight into metal alkoxy halide Lewis acids and are also relevant for group 13 Lewis acids. The results are of direct relevance to nanocrystal reactions with TOPO as a coordinating solvent.

## 4. Experimental

### 4.1. Materials

ZrCl_4_ (99.9%), HfCl_4_ (99.9%), Ti(O^i^Pr)_4_ (98%) and Zr(O*^t^*Bu)_4_ (99.99%) were purchased from Strem Chemicals. TiCl_4_ (99.99%) was bought from ACROS Organics. Acetyl chloride (≥99.0%) was provided by Sigma Aldrich. ZrCl_4_(THF)_2_, TiCl_4_(THF)_2_ and HfCl_4_(THF)_2_ were synthesized following the procedure reported by Manzer *et al.*^56^ ZrBr_4_(THF)_2_ was synthesized in an identical fashion to ZrCl_4_(THF)_2_ (yield = 20%). Zr(O^i^Pr)_4_·^i^PrOH and Hf(O^i^Pr)_4_·^i^PrOH were synthesized following the procedure reported firstly by Bradley *et al.*^57^ and slightly modified by Dhaene *et al.*^58^ Tri-*n*-octylphosphine oxide (Strem Chemicals, 99%) was recrystallized according to the procedure described by Owen *et al.*^59^ while triphenylphosphine oxide (Sigma Aldrich, 98%) was used without further purification. In benzene-d_6_ (Apollo scientific, 99.5 atom%), toluene-d_8_ (VWR, >99.5 atom%), chloroform-d (Eurisotop, 99.5 atom%) and anhydrous pentane (Sigma Aldrich, ≥99%) 10 vol% of activated 4 Å molecular sieves were added and left to stand for 3 days in the glovebox to remove residual water. Toluene and DCM were dried over a solvent system before being transferred into a glovebox. All operations were performed air-free in a nitrogen-filled glovebox.

### 4.2. Methods

#### Preparation of the NMR samples for the spectra in Fig. 2 and 3

ZrCl_3_(O^i^Pr), ZrCl_2_(O^i^Pr)_2_ and ZrCl(O^i^Pr)_3_ were synthesized according to the procedure reported by Bradley *et al.*,^42^ by mixing Zr(O^i^Pr)_4_ and acetyl chloride in different molar ratios. After stirring for 48 hours at room temperature, the solvent is removed and three 0.5 M solutions were prepared in C_6_D_6_ (1 mmol of ZrCl_*x*_(O^i^Pr)_4−*x*_ in 2 mL C_6_D_6_), together with various volumes of a 0.5 M solution of TOPO (773.3 mg, 2 mmol) in C_6_D_6_ (3.12 mL). The different NMR tubes were prepared according to Table 5.

**Table 5:** Details of the preparation of the NMR tubes. For each ZrCl_*x*_(O^i^Pr)_4−*x*_ the 5 NMR have been prepared

*X*_TOPO_	*n*_ZrCl_*x*_(O^i^Pr)_4−*x*__ (mmol)	*n*_TOPO_ (mmol)	*V*_ZrCl_*x*_(O^i^Pr)_4−*x*__ (μL)	*V*_TOPO_ (μL)	*V*_C_6_D_6__ (μL)
0.2	0.12	0.03	240	60	300
0.33	0.1	0.05	200	100	300
0.5	0.075	0.075	150	150	300
0.66	0.05	0.1	100	200	300
0.8	0.03	0.12	60	240	300

The ^1^H NMR and ^31^P NMR spectra are shown in Fig. S8–S10.† All the peaks in the ^31^P NMR spectra were integrated and their sum was normalized for the total amount of TOPO in the solution. For the complexes with two TOPO ligands, the concentration of the complex is half of the bound TOPO concentration. The detailed procedure to obtain the Job Plot is provided in the ESI.†

#### Preparation of the NMR samples for the spectra in Fig. 5

ZrBr_*x*_(O^i^Pr)_4−*x*_ complexes were synthesized by mixing ZrBr_4_(THF)_2_ with Zr(O^i^Pr)_4_ in the right molar ratios and dissolved in C_6_D_6_ to obtain three 0.1 M stock solutions. The NMR tubes are prepared by mixing the 0.1 M C_6_D_6_ solution of ZrBr_*x*_(O^i^Pr)_4−*x*_ (200 μL) with a 0.5 M C_6_D_6_ solution of TOPO (40 μL for 1 equivalent, 80 μL for 2 equivalents) and C_6_D_6_ is added to make up the tubes to a total volume of 400 μL.

ZrCl_*x*_(O^*t*^Bu)_4−*x*_ complexes were synthesized by mixing ZrCl_4_(THF)_2_ with Zr(O^*t*^Bu)_4_ in the right molar ratios and dissolved in C_6_D_6_ to obtain three 0.5 M stock solutions (0.5 mmol ZrCl_*x*_(O^*t*^Bu)_4−*x*_ in 1 mL C_6_D_6_). To improve the solubility of the complexes, 1 equivalent of TOPO is added in each of the three stock solutions (1 mL of 0.5 M C_6_D_6_ solution of TOPO). The NMR tubes for 1 equivalent are prepared by diluting the solution of ZrCl_*x*_(O^*t*^Bu)_4−*x*_ with 1 equiv. TOPO (100 μL) with C_6_D_6_ (400 μL). To prepare the NMR tubes with 4 equiv., the solution of ZrCl_*x*_(O^*t*^Bu)_4−*x*_ with 1 equiv. TOPO (100 μL) is mixed with 0.5 M C_6_D_6_ of TOPO (150 μL) and C_6_D_6_ (250 μL).

HfCl_*x*_(O^i^Pr)_4−*x*_ complexes were synthesized by mixing HfCl_4_ with Hf(O^i^Pr)_4_ in the right molar ratios and dissolved in C_6_D_6_ to obtain three 0.5 M stock solutions (0.5 mmol HfCl_*x*_(O^i^Pr)_4−*x*_ in 1 mL C_6_D_6_). To improve the solubility of the complexes, 1 equivalent of TOPO is added in each of the three stock solutions (1 mL of 0.5 M C_6_D_6_ solution of TOPO). The NMR tubes for 1 equivalent are prepared by diluting the solution of HfCl_*x*_(O^i^Pr)_4−*x*_ with 1 equiv. TOPO (100 μL) with C_6_D_6_ (400 μL). To prepare the NMR tubes with 4 equiv., the solution of HfCl_*x*_(O^i^Pr)_4−*x*_ with 1 equiv. TOPO (100 μL) is mixed with 0.5 M C_6_D_6_ of TOPO (150 μL) and C_6_D_6_ (250 μL).

TiCl_*x*_(O^i^Pr)_4−*x*_ complexes were synthesized by mixing 0.5 M C_6_D_6_ solution of TiCl_4_ with a 0.5 M C_6_D_6_ solution of Ti(O^i^Pr)_4_ in the right volumes. The NMR tubes are prepared by mixing the 0.5 M C_6_D_6_ solution of TiCl_*x*_(O^i^Pr)_4−*x*_ (150 μL for 1 equiv. TOPO, 60 μL for 4 equiv. TOPO) with a 0.5 M C_6_D_6_ solution of TOPO (150 μL for 1 equivalent, 240 μL for 4 equivalents) and C_6_D_6_ is added to make up the tubes to a total volume of 500 μL.

#### Crystal growth of ZrCl_4_(TPPO)_2_

1.5 mL of a 0.1 M toluene solution of TPPO (69.6 mg, 0.25 mmol TPPO in 2.5 mL of toluene) was layered over a 0.1 M DCM solution of ZrCl_4_(THF)_2_ (37.7 mg, 0.1 mmol ZrCl_4_(THF)_2_ in 1 mL of DCM) in a 4 mL vial. White crystals grew after 4 days and a single crystal was chosen for X-ray diffraction, while the rest was dried under vacuum and analyzed by PXRD and ^31^P NMR. Yield of 44%. A detailed characterization consisting of ^1^H NMR and ^31^P NMR (Fig. S2), PXRD (Fig. S4), IR (Fig. S3) and TGA (Table S3) is reported in the ESI.†

#### Crystal growth of HfCl_4_(TPPO)_2_

1.5 mL of a 0.1 M toluene solution of TPPO (69.6 mg, 0.25 mmol TPPO in 2.5 mL of toluene) was layered over a 0.1 M DCM solution of HfCl_4_(THF)_2_ (46.5 mg, 0.1 mmol HfCl_4_(THF)_2_ in 1 mL of DCM) in a 4 mL vial. White crystals grew after 4 days and a single crystal was chosen for X-ray diffraction, while the rest was dried under vacuum and analyzed by PXRD and ^31^P NMR. Yield of 38%. A detailed characterization consisting of ^1^H NMR and ^31^P NMR (Fig. S2), PXRD (Fig. S4), IR (Fig. S3) and TGA (Table S3) is reported in the ESI.†

#### Crystal growth of TiCl_4_(TPPO)_2_

Anhydrous toluene (0.5 mL) was layered over a 0.1 M DCM solution of TiCl_4_(THF)_2_ (33.3 mg, 0.1 mmol TiCl_4_(THF)_2_ in 1 mL DCM) in a 4 mL vial. On top of it, 1 mL of a 0.05 M toluene solution of TPPO (69.6 mg, 0.25 mmol TPPO in 5 mL of toluene) was layered. Yellow crystals grew after 20 hours and a single crystal was chosen for X-ray diffraction, while the rest was dried under vacuum and analyzed by PXRD and ^31^P NMR. Yield of 74%. A detailed characterization consisting of ^1^H NMR and ^31^P NMR (Fig. S2), PXRD (Fig. S4), IR (Fig. S3) and TGA (Table S3) is reported in the ESI.†

#### Crystal growth of ZrBr_4_(TPPO)_2_

ZrBr_4_ (4.1 mg, 0.01 mmol) is dissolved in anhydrous DCM (2 mL) and TPPO (7.0 mg, 0.025 mmol) is added to the solution. Anhydrous pentane (2 mL) was layered over it. A single crystal was selected for X-ray diffraction. Quantitative yield. A detailed characterization consisting of ^1^H NMR and ^31^P NMR (Fig. S2), PXRD (Fig. S4), IR (Fig. S3) and TGA (Table S3) is reported in the ESI.†

#### Crystal growth of ZrBr_4_(THF)_2_ by recrystallization

ZrBr_4_(THF)_2_ is dissolved in anhydrous DCM, and filtrated to remove ZrBr_4_ impurities. ZrBr_4_(THF)_2_ is then precipitated from the solution with anhydrous pentane, vacuum dried and again dissolved in anhydrous DCM. White crystals grew after 2 days through diffusing pentane vapor. A single crystal was selected for X-ray diffraction. A detailed characterization consisting of ^1^H NMR and ^31^P NMR (Fig. S2), PXRD (Fig. S4), IR (Fig. S3) and TGA (Table S3) is reported in the ESI.†

### 4.3. General instrumentation

**Nuclear magnetic resonance (NMR)** measurements were recorded at 298 K on Bruker UltraShield 500 spectrometer operating at a frequency of 500.13 MHz. Regular ^1^H, and ^31^P NMR spectra were acquired using the standard pulse sequences with a 30 degree pulse with a recycle delay of 1.5, and 1.0 second from the Bruker library; zg30, zgpg30 respectively. ^31^P NMR spectra were acquired using inverse gated decoupling and 64 scans and were processed with a line broadening of 5 Hz. All resonances are background-corrected. Chemical shifts (δ) are given in parts per million (ppm), and the residual solvent peak was used as an internal standard (C_6_D_6_: δH = 7.16 ppm, CDCl_3_: δH = 7.26 ppm, tol-d_8_: δH = 2.09 ppm).

#### Single crystal XRD

Single crystal data were collected on STOE STADIVARI diffractometer with a microfocused Cu source. The crystals were kept at a steady *T* = 150 K during data collection. The structures were solved with the ShelXT^60^ solution program using dual methods and by using Olex2^61^ as the graphical interface. The model was refined with ShelXL 2018/3^62^ using full matrix least squares minimization on F^2^.

#### Quantum chemical calculations

All calculations were performed with the B3LYP functional together with the aug-cc-pVDZ basis set for C, H, O, Cl, and P atoms using Gaussian09.^63–66^ The aug-cc-pVDZ pseudopotential and associated basis set of Peterson *et al.* was taken from the Basis Set Exchange and applied to the Zr atoms.^67,68^ This level of theory was previously shown to be accurate for these types of metal complexes.^11^ Only calculations in the gas phase were carried out. To calculate the ^31^P NMR chemical shifts from the optimized structures, we followed the protocol of Willoughby *et al.*^69^

#### Powder X-ray diffraction (PXRD)

PXRD patterns were collected at room temperature in transmission mode using a Stoe Stadi P diffractometer with a micro-focused Cu-Kα-source (*λ* = 1.542 Å) equipped with a DECTRIS MYTHEN 1K detector.

#### Fourier transform infrared spectroscopy (FTIR)

FTIR spectra were recorded air-free in a nitrogen-filled glovebox in transmission mode on a Bruker Alpha II FTIR-Spectrometer. The measured pellets were prepared air-free by mixing the sample with potassium bromide and compressing them.

#### Thermogravimetric analysis (TGA)

TGA was performed on a TGA5500 (TA instruments) instrument. The samples were heated to 800 °C at a ramping rate of 5 °C min^−1^. At the end an isotherm of 15 min is given to ensure that all the organics are burned out.

## Conflicts of interest

There are no conflicts to declare.

## Supplementary Material

DT-053-D4DT01299B-s001

DT-053-D4DT01299B-s002

DT-053-D4DT01299B-s003
