# Supplementary material for: Complexation and disproportionation of group 4 metal (alkoxy) halides with phosphine oxides
Source: Dalton Trans. 2024 May 28;53(23):9862–73. doi: 10.1039/d4dt01299b (PMC11163947; doi:10.1039/d4dt01299b)
Supplement: DT-053-D4DT01299B-s002 [file DT-053-D4DT01299B-s002.pdf]

Electronic Supplementary Material (ESI) for Dalton Transactions.
This journal is © The Royal Society of Chemistry 2024

Supporting Information: Complexation and Disproportionation of Group 4 Metal (Alkoxy) Halides with Phosphine Oxides

Carlotta Seno, Rohan Pokratath, Ajmal Roshan Unniram Parambil, Dietger Van den Eynden, Evert Dhaene, Alessandro Prescimone, and Jonathan De Roo*

Department of Chemistry, University of Basel, Mattenstrasse 22, 4058 Basel, Switzerland

E-mail: Jonathan.DeRoo@unibas.ch

Crystallographic data

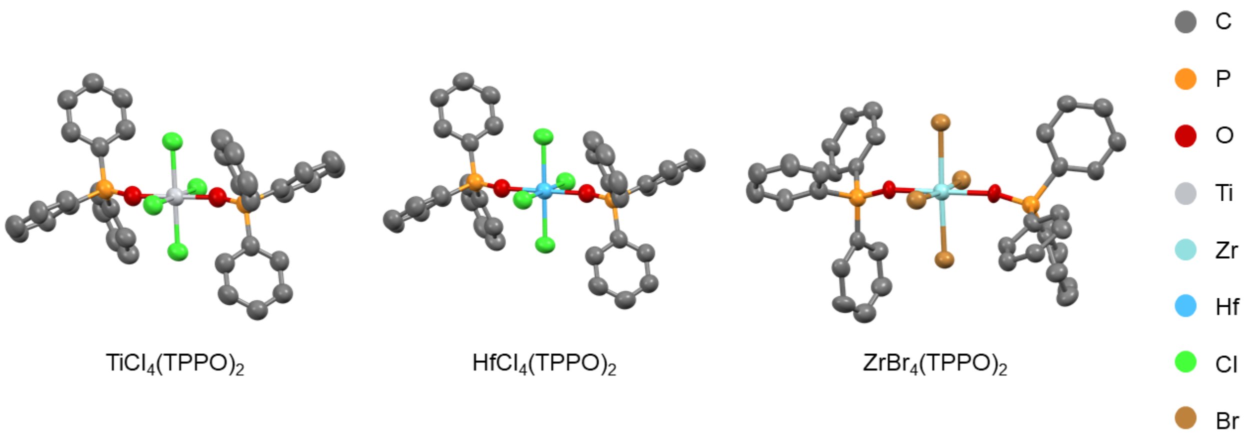

Figure S1: Crystal structures of the synthesized metal halide complexes captured with Single Crystal XRD. The hydrogen atoms are omitted for clarity.

Table S1: Comparison of bond lengths in the adducts of metal halides with Lewis bases (THF or TPPO).

	M-X [Å]	M-O [Å]		M-X [Å]	M-O [Å]
cis-$TiF_4(THF)_2$[S1,b]	1.789(1), 1.792(1)	2.110(2)	*cis*-$TiF_4(TPPO)_2$[S1,b]	1.805, 1.840	2.039
	1.809(1), 1.810(1)	2.132(1)			
cis-$TiCl_4(THF)_2$[S2,b]	2.2514(5), 2.2617(6)	2.108(1)			
	2.2918(5), 2.2945(5)	2.110(1)			
trans-$TiCl_4(THF)_2$[S3,b]	2.408, 2.4018	2.083	*trans*-$TiCl_4(TPPO)_2$[S4,c]	2.3283, 2.3336	1.923
			trans-$TiCl_4(TPPO)_2$[a]	2.3276(11) 2.3361(13)	1.932(4)
			trans-$ZrF_4(TPPO)_2$[S5,b]	1.975, 1.987	2.116
cis-$ZrCl_4(THF)_2$[S6,c]	2.389(4), 2.397(4)	2.228(7)	*trans*-$ZrCl_4(TPPO)_2$[a]	2.4472(17), 2.4581(18)	2.062(5)
	2.422(3), 2.425(3)	2.237(8)			
trans-$ZrBr_4(THF)_2$[a]	2.5816(18), 2.5830(17)	2.178(10)	*trans*-$ZrBr_4(TPPO)_2$[a]	2.588(3), 2.622(3)	2.086(13)
				2.629(3), 2.643(3)	2.103(13)
cis-$HfCl_4(THF)_2$[S7,c]	2.363(4), 2.377(4)	2.194(8)	*trans*-$HfCl_4(TPPO)_2$[a]	2.4350(19), 2.445(2)	2.065(5)
	2.388(4), 2.401(3)	2.197(8)			

[a] this work, measured at 150K, [b] measured between 100 and 123K, [c] measured at room temperature.

Table S2: Crystallographic parameters for the reported crystal structures.

Structure	$ZrCl_4(TPPO)_2$	$TiCl_4(TPPO)_2$	$HfCl_4(TPPO)_2$	$ZrBr_4(TPPO)_2$	$ZrBr_4(THF)_2$
CCDC	2219601	2219602	2219605	2219604	2219606
Formula	$C_{36}H_{30}Cl_4O_2P_2Zr$	$C_{36}H_{30}Cl_4O_2P_2Ti$	$C_{36}H_{30}Cl_4HfO_2P_2$	$C_{36}H_{30}Br_4O_2P_2Zr$	$C_8H_{16}Br_4O_2Zr$
$D_{calc.}$/ g cm^{-3}	1.481	1.439	1.647	1.775	2.41
μ/mm^{-1}	6.427	6.096	9.344	8.743	17.995
Formula Weight/ g mol^{-1}	789.56	746.24	876.83	967.4	555.07
Colour	colourless	yellow	colourless	colourless	colourless
Shape	block-shaped	plate-shaped	block-shaped	block-shaped	plate-shaped
Size/mm^3	0.17×0.16×0.15	0.23×0.183×0.1	0.18×0.143×0.11	0.80×0.33×0.08	0.26×0.18×0.07
T/K	150	150	150	150	150
Crystal System	triclinic	triclinic	triclinic	monoclinic	triclinic
Flack Parameter				0.04(4)	
Hooft Parameter				0.085(7)	
Space Group	P-1	P-1	P-1	$P2_1$	P-1
a/Å	9.4661(4)	9.4279(3)	9.4917(3)	9.9980(3)	7.1420(7)
b/Å	9.5612(4)	9.5623(3)	9.5535(3)	15.5366(5)	7.5304(8)
c/Å	10.5356(4)	10.3638(3)	10.5075(3)	11.8659(3)	8.0799(8)
α/°	101.126(3)	102.086(2)	101.253(2)	90	87.085(8)
β/°	107.899(3)	108.255(2)	108.000(2)	100.875(2)	68.311(8)
γ/°	92.429(3)	92.847(2)	92.173(3)	90	71.788(8)
V/Å^3	885.05(6)	860.92(5)	883.81(5)	1810.09(9)	382.53(7)
Z	1	1	1	2	1
Wavelength/Å	1.54186	1.54186	1.54186	1.54186	1.54186
Radiation type	Cu K_α	Cu K_α	Cu K_α	Cu K_α	Cu K_α
Θ_{min}/°	4.518	4.625	4.746	3.793	7.312
Θ_{max}/°	72.804	72.884	73.121	72.911	73.062
Measured Refl's.	16749	15120	14940	23579	6412
Indep't Refl's	3432	3380	3442	6613	1496
Refl's I≥ 2 σ(I)	3120	3038	3300	5850	1079
R_{int}	0.0353	0.0453	0.0253	0.0446	0.0482
Parameters	206	206	206	407	70
Restraints	0	0	0	433	60
Largest Peak	1.15	0.72	1.2	1.772	1.089
Deepest Hole	-1.27	-0.72	-1.42	-1.607	-0.914
GooF	0.903	0.916	1.036	1.091	1.1
wR_2 (all data)	0.1820	0.1991	0.1588	0.2455	0.1949
wR_2	0.1668	0.1865	0.1514	0.2181	0.1752
R_1 (all data)	0.0979	0.0925	0.0710	0.1117	0.1156
R_1	0.0840	0.0832	0.0661	0.0938	0.0846

Characterization of Single Crystals

^{1}H NMR and ^{31}P NMR

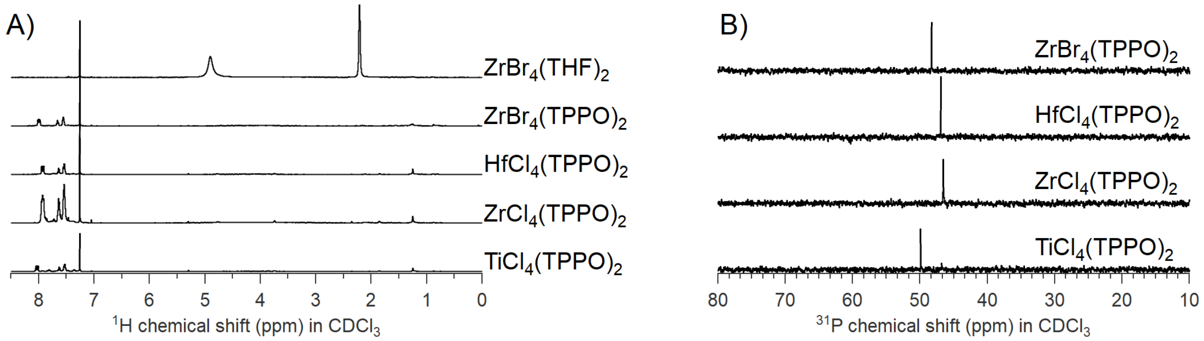

Figure S2: Room temperature A) ^{1}H NMR B) ^{31}P NMR spectra of the newly reported compounds. Note that all compounds are very poorly soluble.

Thermogravimetric Analysis (TGA)

Table S3: TGA Analysis of the newly synthesized compounds. The larger deviation from the ideal weight loss is likely due to the formation of zirconium phosphate. The complete weight loss for the titanium complex is attributed to the volatile nature of $TiCl_4$.

Starting compound	MW g mol^{-1}	Residual weight TGA	Weight loss	Hypothesized residual species	MW residual g mol^{-1}	theoretical residual weight	Error
$TiCl_4(TPPO)_2$	746.24	**0.02 %**	99.98%	TiO_2	79.87	**9.1%**	455 %
$ZrCl_4(TPPO)_2$	789.56	**16.8 %**	83.2%	ZrO_2	123.22	**15.6%**	7 %
$HfCl_4(TPPO)_2$	876.83	**24.2 %**	75.8%	HfO_2	210.49	**24.0%**	1 %
$ZrBr_4(TPPO)_2$	967.40	**13.9 %**	86.1%	ZrO_2	123.22	**12.7%**	9 %
$ZrBr_4(THF)_2$	555.07	**23.1 %**	76.9%	ZrO_2	123.22	**22.2%**	4 %

Infrared Spectroscopy

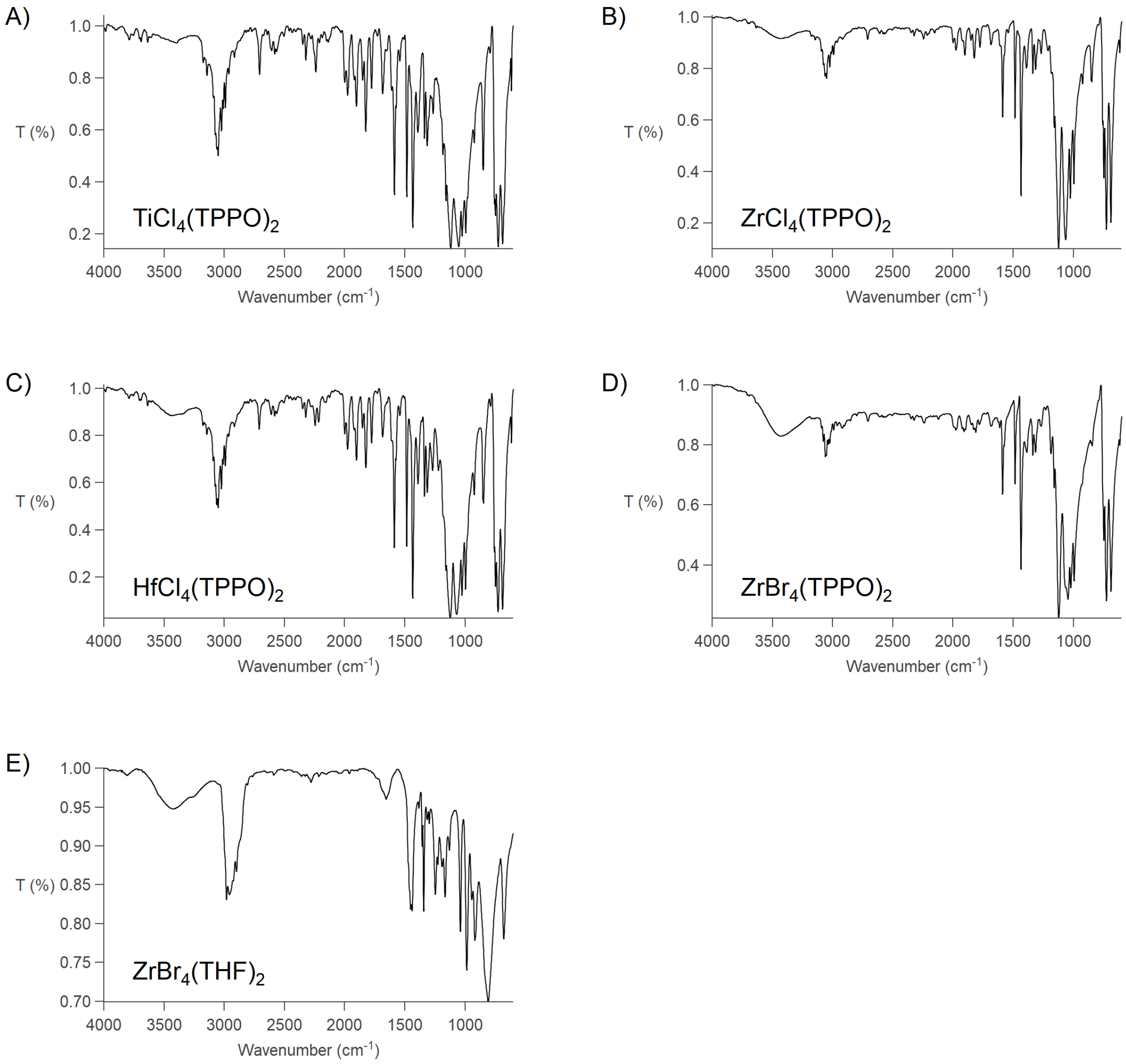

Figure S3: IR spectra acquired and baseline corrected with the software OPUS 8.5 of the newly reported complexes A) $TiCl_4(TPPO)_2$ B) $ZrCl_4(TPPO)_2$ C) $HfCl_4(TPPO)_2$ D) $ZrBr_4(TPPO)_2$ E) $ZrBr_4(THF)_2$

Powder XRD

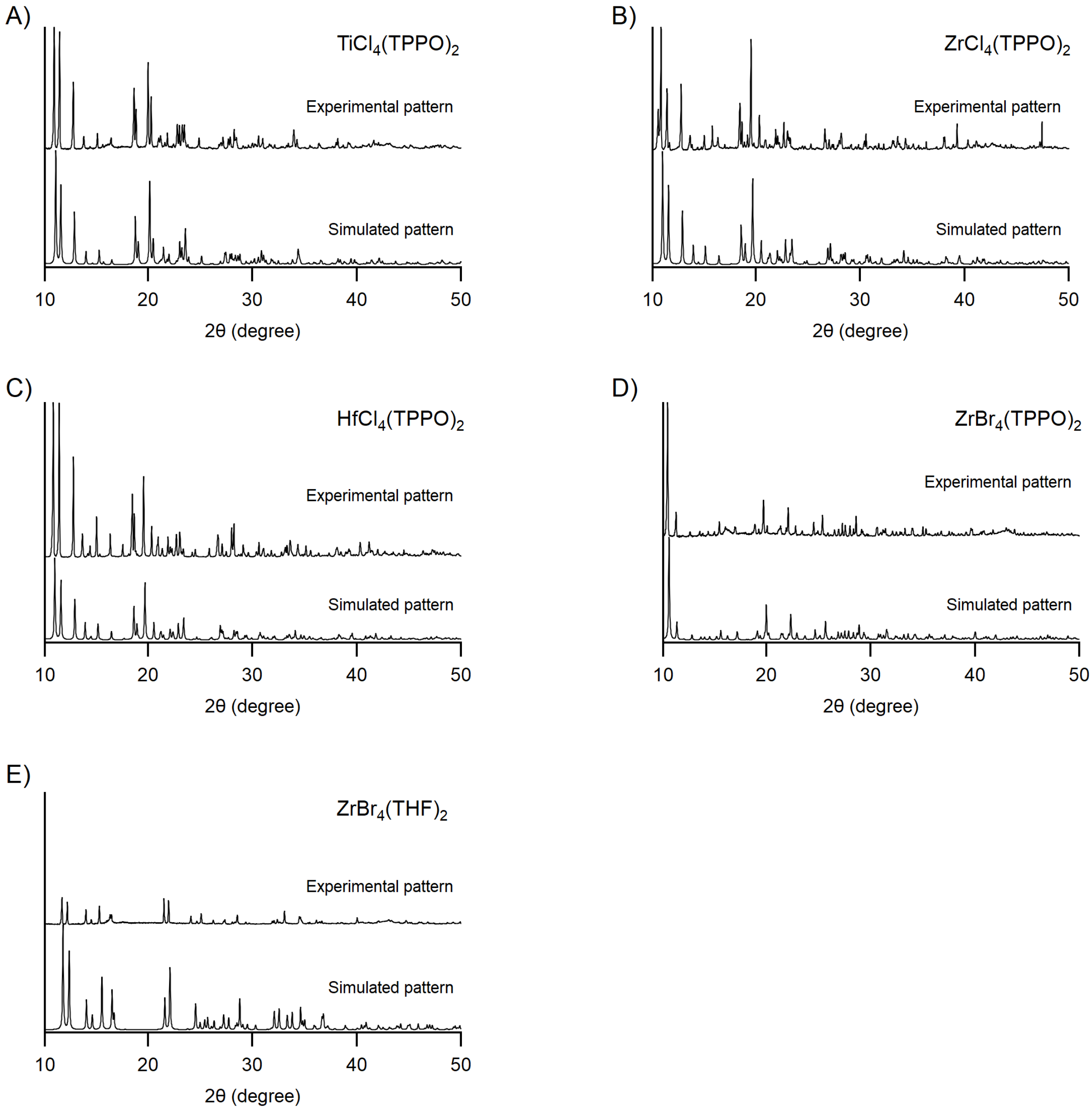

Figure S4: Powder XRD patterns simulated (with λ= 1.542 Å) from the single crystal data and of the bulk material of the newly reported compounds A) $TiCl_4(TPPO)_2$ B) $ZrCl_4(TPPO)_2$ C) $HfCl_4(TPPO)_2$ D) $ZrBr_4(TPPO)_2$ E) $ZrBr_4(THF)_2$.

Confirmation of the chemical shifts for $MCl_4(TOPO)_2$ complexes

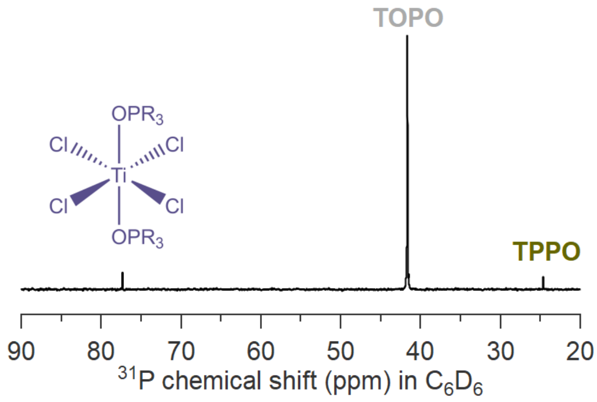

Figure S5: ^{31}P NMR spectrum at room temperature of $TiCl_4(TPPO)_2$ with 1 equivalent of TOPO with respect to the metal.

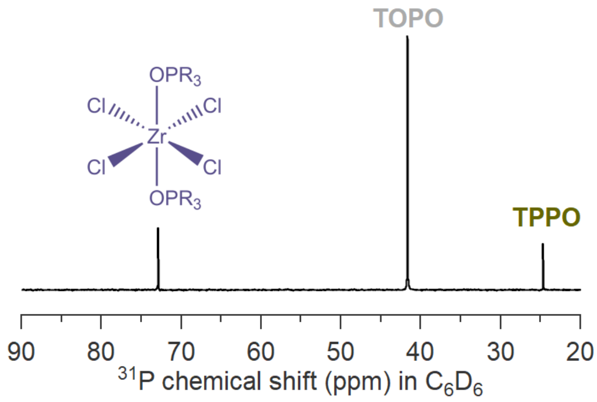

Figure S6: ^{31}P NMR spectrum at room temperature of $ZrCl_4(TPPO)_2$ with 1 equivalent of TOPO with respect to the metal.

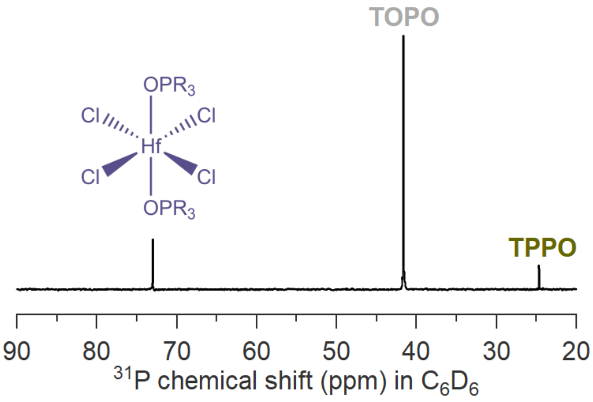

Figure S7: ^{31}P NMR spectrum at room temperature of $HfCl_4(TPPO)_2$ with 1 equivalent of TOPO with respect to the metal.

Quantitative fit of the Job plot

We pursued the steady-state fitting of the Job plot with the software Copasi. For each Job plot, i.e. for each zirconium chloroalkoxide species, we created an input file (.txt) containing the equilibrium concentrations for the five different mole fractions in the Job plot, (reported in Tables S4, S5, S6). In the input file, the first line describes the identity of the species and the other five report the concentration in mol/L for each equilibrium condition. Each equilibrium corresponds to a different ^{31}P NMR spectrum (see Figures S8B, S9B and S10B). The concentration of all species was determined as described below.

Table S4: Input file used to fit the Job Plots of $ZrCl_3(O^iPr)$ in Figure 3. The concentrations of the species considered in the fittings are reported in mol/L. TOPO is abbreviated with T.

Equilibrium concentration (mol/L)				Initial concentration (mol/L)	
$ZrCl_4T_1$	$ZrCl_4T_2$	$ZrCl_3(O^iPr)T_2$	free T	$ZrCl_3(O^iPr)$	free T
0.01550	0.01208	0	0	0.18967	0.03967
0.00800	0.02900	0	0	0.14933	0.06600
0	0.03800	0.01283	0	0.10167	0.10167
0	0.00100	0.05467	0.03150	0.05950	0.14283
0	0.00075	0.03183	0.11783	0.03300	0.18300

Table S5: Input file used to fit the Job Plots of $ZrCl_2(O^iPr)_2$ in Figure 3. The concentrations of the species considered in the fittings are reported in mol/L. TOPO is abbreviated with T.

Equilibrium concentration (mol/L)				Initial concentration (mol/L)	
$ZrCl_4T_2$	$ZrCl_3(O^iPr)T_2$	$ZrCl_2(O^iPr)_2T_2$	free T	$ZrCl_2(O^iPr)_2$	free T
0.00508	0.00333	0	0	0.18342	0.01683
0.00325	0.01825	0	0	0.14650	0.04300
0	0.04183	0.01425	0	0.11858	0.11217
0	0.00242	0.05675	0.03450	0.07642	0.15283
0	0.00175	0.03258	0.12217	0.04542	0.19083

Table S6: Input file used to fit the Job Plots of $ZrCl(O^iPr)_3$ in Figure 3. The concentrations of the species considered in the fittings are reported in mol/L. TOPO is abbreviated with T.

Equilibrium concentration (mol/L)				Initial concentration (mol/L)	
$ZrCl_3(O^iPr)T_2$	$ZrCl_2(O^iPr)_2T_2$	$ZrCl(O^iPr)_3T_2$	free T	$ZrCl(O^iPr)_3$	free T
0.00625	0.00133	0	0	0.16517	0.01517
0.00592	0.01308	0	0	0.12133	0.03800
0	0.03917	0.00375	0.00833	0.09417	0.09417
0	0.01767	0.02692	0.06133	0.06717	0.15050
0	0.01033	0.02158	0.13117	0.0450	0.19500

Firstly, all the resonances in the ^{31}P NMR spectra are integrated to determine the equilibrium concentrations. The sum of integrals is normalized for the total concentration of TOPO in the solution (see Table 5 in the main text). The majority of the resonances were assigned as shown in Figures S8B, S9B and S10B. However a few small peaks remain unassigned (indicated by a star symbol). For the assigned complexes with two TOPO ligands per zirconium, the concentration of the complex is half of the integral. For the free TOPO resonance and the $ZrCl_4$(TOPO) complex, the concentration is equal to the integral.

In the input file, we also tell the COPASI program the initial concentration of the reagents (here: TOPO and $ZrCl_x(O^iPr)_{4-x}$), before equilibration. In principle, these columns would correspond to the values in Table 5 in the main text. However, a minority of resonances in the ^{31}P NMR spectra remained unassigned and thus we slightly corrected the initial concentrations. The corrected initial concentration of TOPO is the sum of the integrals of the assigned peaks in the spectrum. The other peaks represented also species with bound

TOPO, which is therefore unavailable for the equilibria that we are modeling. Also the initial $ZrCl_x(O^iPr)_{4-x}$ concentration is corrected, by assuming that there is one TOPO molecule bound per Zr atom in the unassigned species. Therefore, the corrected initial concentration of $ZrCl_x(O^iPr)_{4-x}$ is equal to the theoretical concentration (Table 5) minus the sum of all unassigned species in the ^{31}P NMR spectrum. These corrections lead typically to 5-10% difference with the theoretical value.

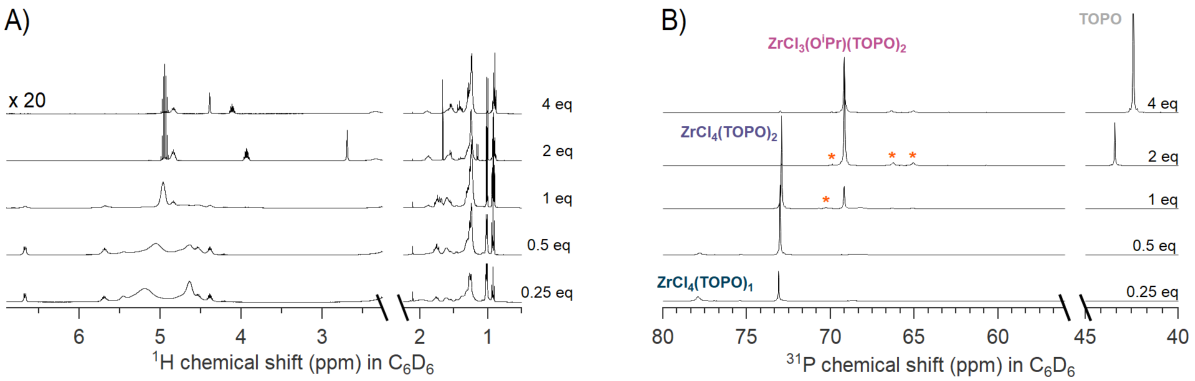

Figure S8: A)^{1}H NMR and B) ^{31}P NMR spectra of $ZrCl_3(O^iPr)$ with different amounts of TOPO. From the ^{31}P NMR spectra the Job Plot has been calculated.

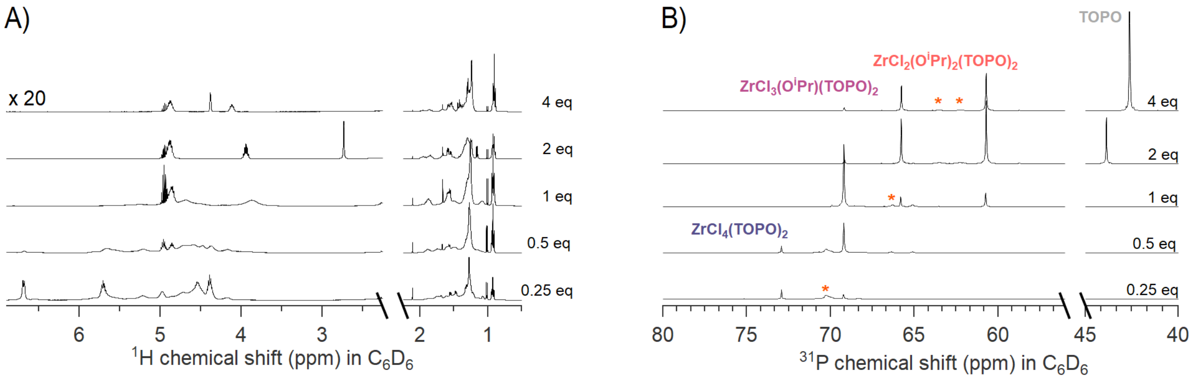

Figure S9: A)^{1}H NMR and B) ^{31}P NMR spectra of $ZrCl_2(O^iPr)_2$ with different amounts of TOPO. From the ^{31}P NMR spectra the Job Plot has been calculated.

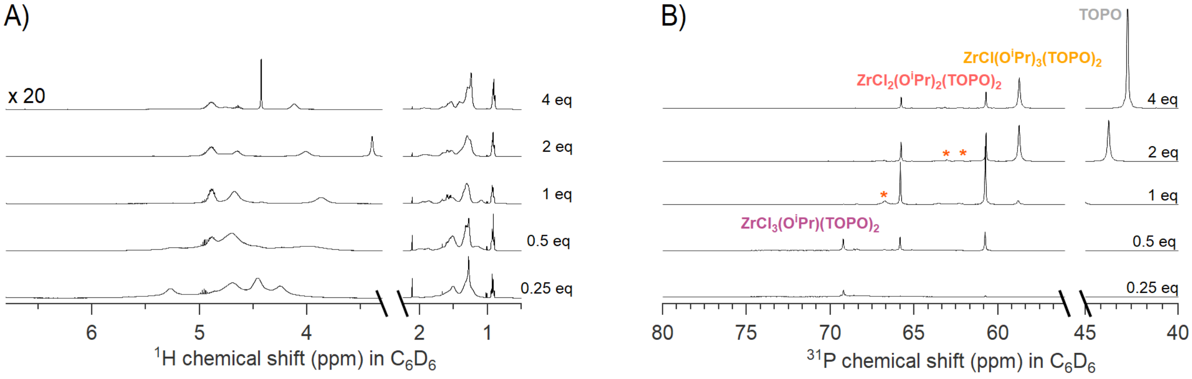

Figure S10: A)^{1}H NMR and B) ^{31}P NMR spectra of $ZrCl(O^{i}Pr)_3$ with different amounts of TOPO. From the ^{31}P NMR spectra the Job Plot has been calculated.

Complexes with TEPO

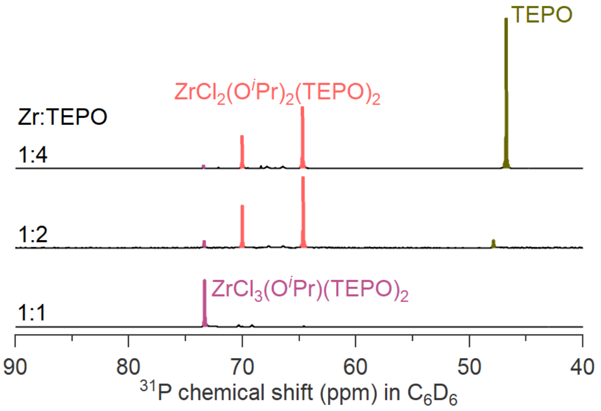

Figure S11: ^{31}P NMR spectra at room temperature of $ZrCl_2(O^{i}Pr)_2$ titrated with TEPO. The ratio of Zr to TEPO is indicated in the figures.

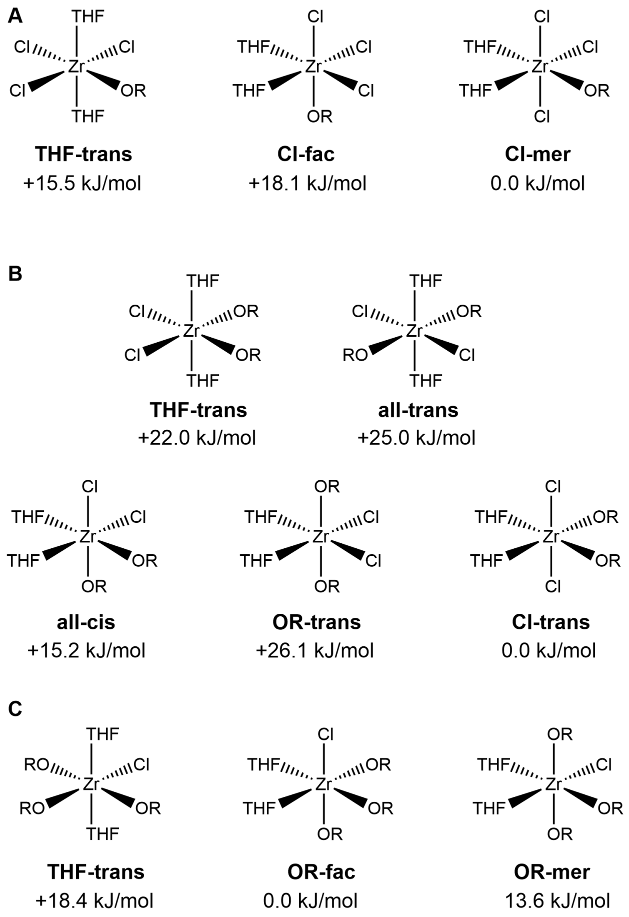

Figure S12: The different possible isomers for the $ZrCl_x(O^iPr)_{4-x}$ (x=1-3) complexes with THF. The relative energy compared to the most stable isomer is indicated.

Effect of THF on $ZrCl_3(O^iPr)$

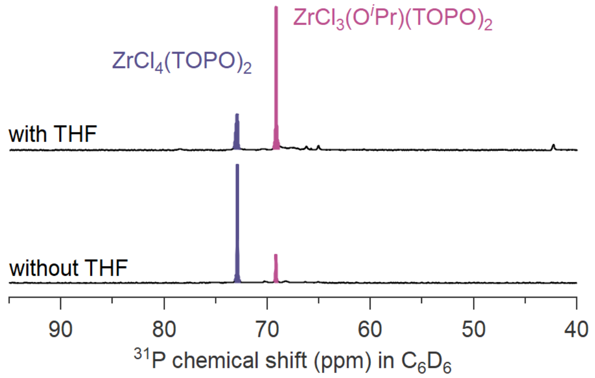

Figure S13: ^{31}P NMR spectra at room temperature of $ZrCl_3(O^iPr)$ with 1 equivalents of TOPO once synthesized from $Zr(O^iPr)_4$ and acetyl chloride, once mixing $ZrCl_4(THF)_2$ and $Zr(O^iPr)_4$.

Generalization

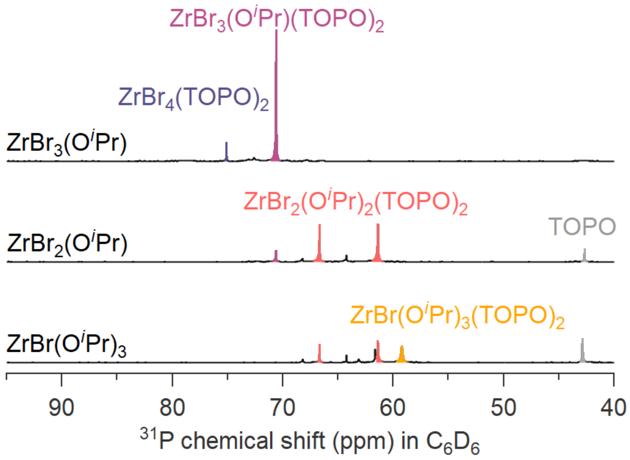

Figure S14: ^{31}P NMR spectra at room temperature of zirconium isopropoxy bromide complexes with 2 equivalents of TOPO.

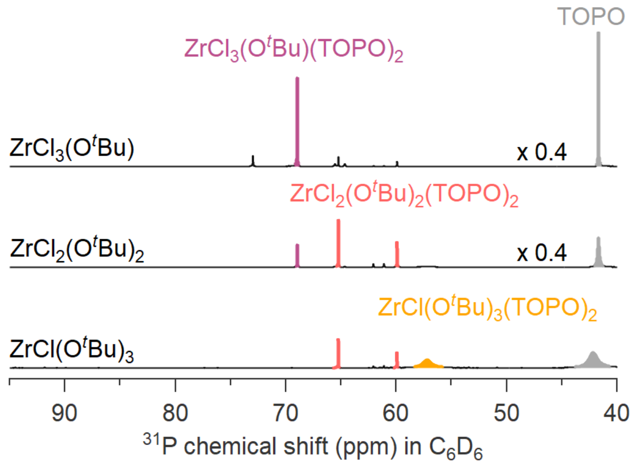

Figure S15: ^{31}P NMR spectra at room temperature of zirconium tertbutoxy chloride complexes with 4 equivalents of TOPO.

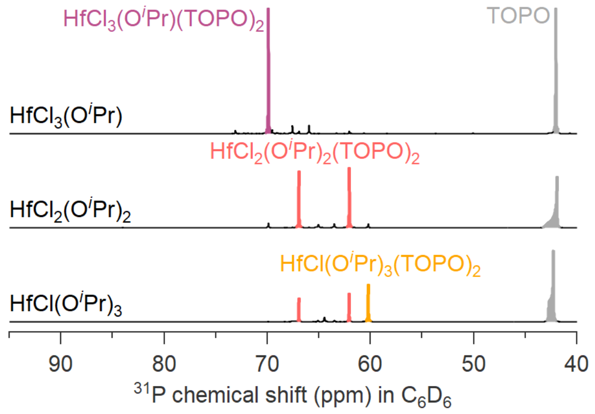

Figure S16: ^{31}P NMR spectra at room temperature of hafnium isopropoxy chloride complexes with 4 equivalents of TOPO.

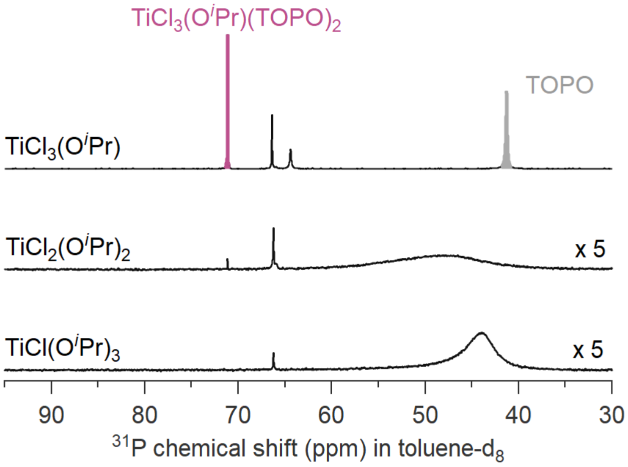

Figure S17: ^{31}P NMR spectra at room temperature of titanium isopropoxy chloride complexes with 4 equivalents of TOPO.

References

(S1) Jura, M.; Levason, W.; Petts, E.; Reid, G.; Webster, M.; Zhang, W. Taking TiF_4 complexes to extremes-the first examples with phosphine co-ligands. *Dalton Transactions* **2010**, *39*, 10264–10271.

(S2) Nieger, M.; Szarvas, L.; Gudat, D. *CSD Communication* **2022**,

(S3) Calderazzo, F.; Englert, U.; Pampaloni, G.; Volpe, M. Redox reactions with bis (η6-arene) derivatives of early transition metals. *Journal of Organometallic Chemistry* **2005**, *690*, 3321–3332.

(S4) Li, M.-F.; Shan, Y.-K. Crystal structure of trans-bis (triphenylphospine oxide) tetrachlorotitanum (IV), $TiCl_4[(C_6H_5)_3PO]_2$. *Zeitschrift für Kristallographie-New Crystal Structures* **2006**, *221*, 41–42.

(S5) Benjamin, S. L.; Levason, W.; Pugh, D.; Reid, G.; Zhang, W. Preparation and structures of coordination complexes of the very hard Lewis acids ZrF_4 and HfF_4. *Dalton Transactions* **2012**, *41*, 12548–12557.

(S6) Eberle, M.; Röhr, C. Tetrachlorobis (tetrahydrofuran-O) zirconium (IV). *Acta Crystallographica Section C: Crystal Structure Communications* **1996**, *52*, 566–568.

(S7) Duraj, S.; Towns, R.; Baker, R.; Schupp, J. Structure of cis-tetrachlorobis (tetrahydrofuran) hafnium (IV). *Acta Crystallographica Section C: Crystal Structure Communications* **1990**, *46*, 890–892.
